# mGem: Transmission and exposure risks of dairy cow H5N1 influenza virus

**DOI:** 10.1128/mbio.02944-24

**Published:** 2025-02-11

**Authors:** A. J. Campbell, Kayla Brizuela, Seema S. Lakdawala

**Affiliations:** 1 Department of Microbiology and Immunology, Emory University, Atlanta, Georgia, USA; The Ohio State University, Columbus, Ohio, USA

**Keywords:** influenza, dairy cow, transmission, H5N1

## Abstract

In March 2024, highly pathogenic H5N1 was detected in dairy cows; as of 12 December 2024, it had spread to over 800 herds in 16 states. The ongoing outbreak is a public health crisis affecting both humans and animals, as interspecies transmission has emerged as a common characteristic of this virus. As of 12 December 2024, >30 humans have been infected in the United States related to dairy cow exposure. In this mGem, we discuss transmission modalities between cows within herds, the spread of the virus between dairy farms, and exposure risks for humans. We also highlight major gaps in knowledge constituting barriers to our ability to effectively control the spread of H5N1 in dairy cows and reduce the risks to humans.

## PERSPECTIVE

**Fig 1:**
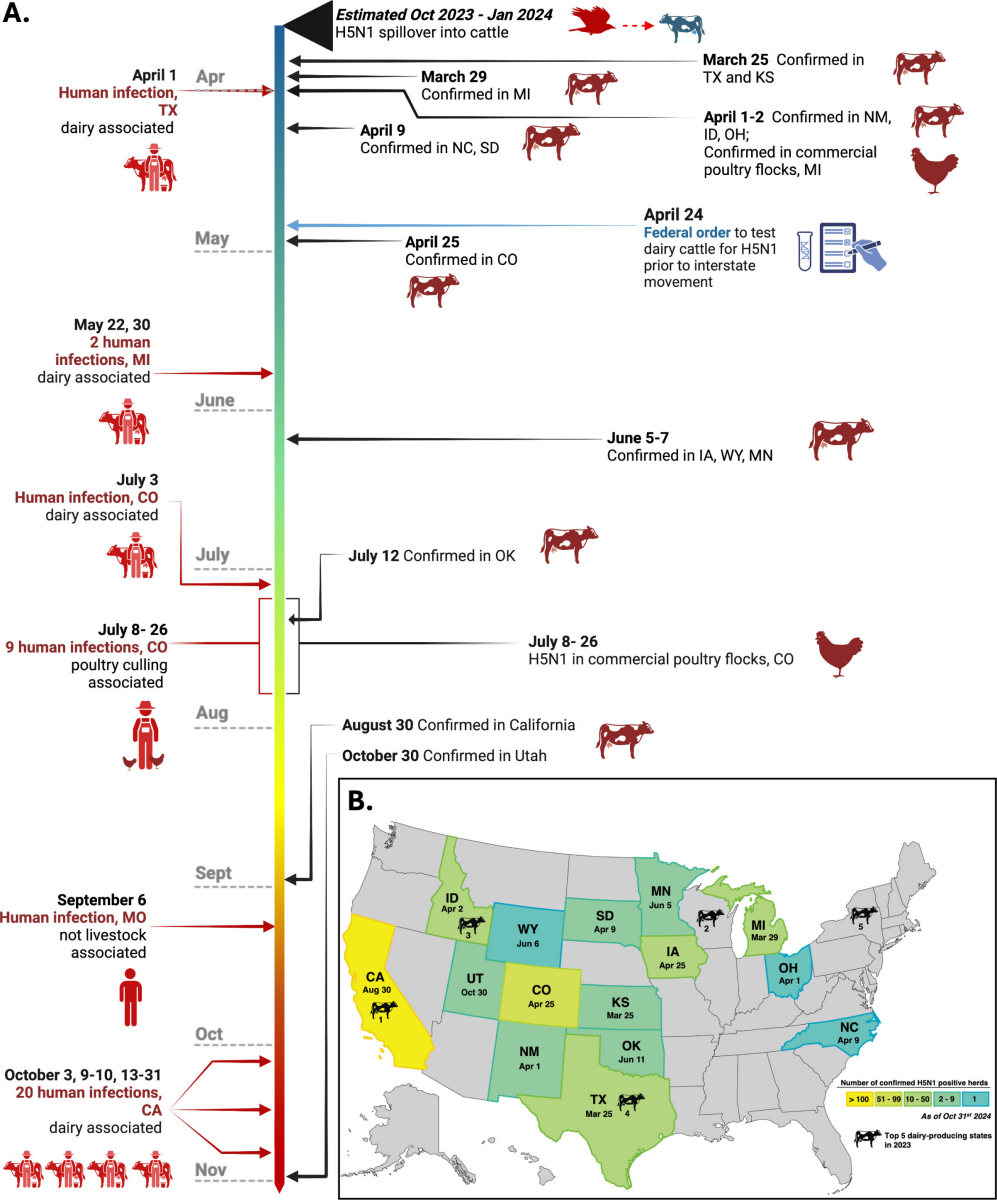
Spatiotemporal overview of the HPAI H5N1 outbreak in the United States. (**A**) Timeline of cow H5N1 outbreak events in the United States from late 2023 until 31 October 2024. Confirmations of virus positivity in new states on the right, black; confirmed human cases on the left, red; federal orders, blue. Events on the timeline were curated from the following references (9, 10, 13, 15–36). (**B**) Heatmap of U.S. states with H5N1 positive herds over the total course of the outbreak until 31 October 2024. State abbreviations and date of the first positive confirmation listed. CA, WI, ID, TX, and NY are the top five dairy producers in the United States and are demarcated by a cow icon. Images created using BioRender.

Highly pathogenic avian influenza (HPAI) H5N1 virus of the 2.3.4.4b clade has been circulating in North America since 2022 (1–3). On 25 March 2024, a strain in this clade, genotype B3.13, was confirmed to be infecting dairy cows in Texas and Kansas (Fig. 1A). Since then, dairy herds in several other states tested positive for this strain and spillover into dairy workers has followed (4–7). Infections of other domestic and peridomestic animals, such as cats and raccoons, have also occurred on the same premises containing infected cows (7, 8). Notably, the spread of cow H5N1 to poultry flocks has resulted in the culling of millions of birds and the infection of workers involved in culling operations on multiple occasions (9). As of 12 December 2024, over 800 dairy herds in 16 states have had confirmed infections, including three of the five biggest dairy-producing states (Fig. 1B) (10, 11). At least 58 known human cases of H5N1 including those exposed to dairy cows, poultry, or unknown exposure source have been reported by the CDC as of 12 December 2024 (12). Disturbingly, in September, cow H5N1 infections were confirmed in two housemates in Missouri without livestock contact (13, 14). Understanding how cow H5N1 transmits within and between farms is critical for curtailing the outbreak. Below, we discuss knowledge regarding the spread of cow H5N1 between farms, between cows, and the risk to humans.

## TRANSMISSION BETWEEN FARMS

Virus spread between farms has been definitively linked to the shipment of infected cattle, while shared equipment, vehicles, and personnel have also been identified as potential sources for the introduction of virus onto a premises (15). In a USDA survey of affected premises, >50% of surveyed dairies shared vehicles, >20% shared personnel, and >60% shared support services (veterinarians, hoof trimmers, etc.) with at least one other farm (37). Therefore, shared personnel or shared equipment likely contribute to the transmission of H5N1 between farms, especially in herds without known import of cows. Importantly, multiple introductions of H5N1 from avian species into cattle are unlikely given the low diversity of the H5N1 sequences from cattle herds, suggesting a single introduction that continues to propagate (16, 38). More data on the trajectories of farm infections are needed to accurately resolve the sources of transmission.

### Gaps

How a cow becomes infected by a person or contaminated equipment is currently unclear. There is evidence that H5N1 infections in dairy workers are underreported (39), so infected individuals in close contact with cows and/or milk could transmit the virus to animals at different sites. Additional surveillance and information on the trajectories of farm infections and the seropositivity of individuals who travel between farms are needed to accurately resolve the sources of transmission.

## TRANSMISSION OF H5N1 BETWEEN COWS

Infection of dairy cows with HPAI H5N1 can lead to an array of symptoms ranging from mild to severe. Commonly, infected cows are lethargic with decreased appetite. According to a recent USDA survey of infected premises, clinical signs in affected cows typically lasted from 6 to 17 days (37). Lactating cows experience a dramatic reduction in milk production, reduced appetite and rumen motility, and often produce milk that is clotted and of abnormal consistency and color (referred to as mastitis) (7, 40). H5N1 viral RNA has been detected in nasal swabs, urine, and milk of infected animals (40, 41), with high levels of infectious virus (10^4^–10^8^ PFU/mL) observed in expressed milk (8, 42). Many cows survive the infection with low reported lethality (37). However, if symptoms were severe, farmers occasionally chose to humanely euthanize animals (37). In a controlled experimental infection of lactating cows, the milk production within H5N1-infected cows remained at 71%–77% of baseline milk levels 23 days postinfection (40). Similar to field observations, infected calves showed no clear signs of illness (40, 41). The lack of severe clinical disease or mortality in cows has made it challenging to identify infected cows within a herd, while failure to swiftly isolate infected animals allows for continued virus spread between cows on the same farm.

**Fig 2:**
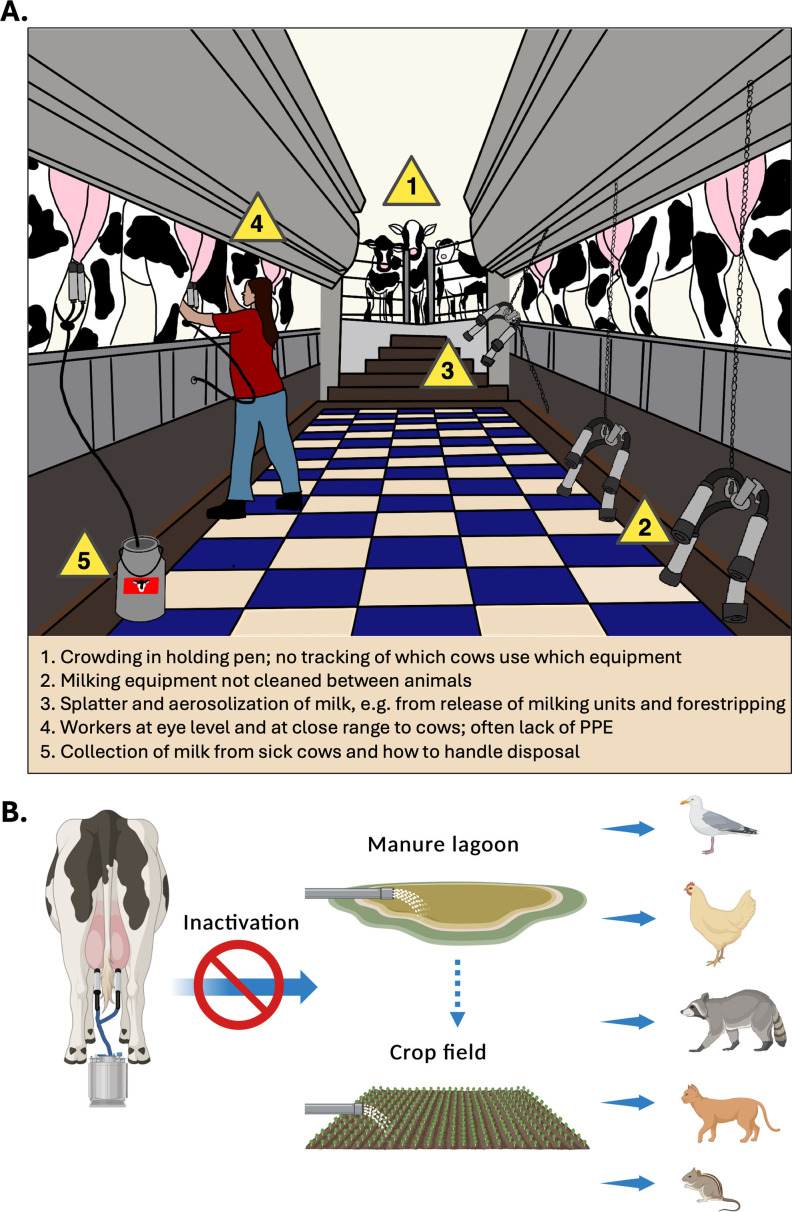
Risks of H5N1 transmission related to milking parlors and milk waste stream. (A) Schematic of a typical large-scale milking parlor; caution symbols indicate described transmission risks either between cows (numbers 1 and 2) or to dairy farm workers (numbers 3–5). PPE, personal protective equipment. (**B**) Waste stream for contaminated or unsaleable milk, which could infect other animals if not inactivated after collection. Images created using BioRender.

Potential modes of cow-to-cow transmission include direct contact with contaminated milking equipment, aerosolization and respiration of virus-laden milk during the milking process, and close proximity between cows within their holding pens (Fig. 2A). Given minimal detection of viral RNA in nasal swabs and high levels of infectious virus in milk, transmission between cows likely involves contaminated milk. The milking process, wherein many lactating cows share the same equipment, is a likely avenue through which further H5N1 infections are propagated within a herd (Fig. 2) (40, 43). Notably, while the teats of cows are cleaned and treated with an anti-mastitis solution prior to milking, no treatment or disinfectant is applied to the milking equipment. Raw milk from infected cows harbors infectious virus (8, 42), and cow H5N1 virus is capable of persisting in milk and on milking unit surfaces across a range of relative humidities for at least an hour (43). If the average time to milk a cow is 4 to 6 min (44), over the course of an hour, multiple cows using the same contaminated equipment would be at risk (Fig. 2A). There is epidemiological data to suggest transmission of bacterial pathogens via contaminated milking equipment (45–47) and a similar mechanism could facilitate transmission of H5N1 between cows. Automatic milking units function via the application of negative pressure and physically squeezing the teats, which promotes milk flow. It is possible that aberrations to this pressure regime, or an imperfect seal between the milking unit and the teat, might occasionally induce upward movement of milk back into the teat canal. Importantly, dairy cow mammary glands contain both α2,6 and α2,3 sialic acids receptors, and this tissue site seems to be highly susceptible to viral replication (48, 49). Indeed, cows inoculated with cow H5N1 via the intramammary route contained virus in their mammary glands and produced milk containing infectious virus (40), highlighting the importance of this site for virus infection and providing an explanation for the high titers observed in milk.

All lactating cows must be milked every day for the health of the animal, including those identified as sick. For consumer safety, standard practice on dairy farms is for cows on antibiotics or those with mastitis to be milked separately such that the sick milk is separated from bulk milk in various ways depending on dairy size and capacity (Fig. 2A). Importantly, sick cows that are on antibiotics or have mastitis could also harbor undetected H5N1, and while they would be milked separately, the disposal practices involving this sick milk could become potential routes for H5N1 transmission on farms. The USDA recommends heat treating the sick milk similarly to pasteurization (50), but it is unclear how many farms are capable of this intervention. The sick milk waste stream is typically diverted to manure lagoons, the majority of which are open to the environment and potentially accessible to peridomestic wildlife (Fig. 2B), or bottle-fed to calves. Both avenues of disposal represent risks to continue the spread of H5N1 in nature. Consumers should note that milk from cows with subclinical presentation of H5N1 infection, i.e., not presenting with mastitis or other symptoms, could still contain a virus that would flow into normal milk processing pipelines.

### Gaps

As of December 2024, the number of cows infected with H5N1 on a given positive farm is largely unknown. The lack of cow-specific data prevents accurate assessment of the efficiency of H5N1 transmission between cows and the primary routes of infection on a farm. The contribution of respiratory transmission cannot be ruled out without further information about the longitudinal kinetics of H5N1 infections within individual cows and the dynamics of cow-to-cow transmission on a farm. In addition, epidemiological or experimental data on the role of contaminated milking equipment in facilitating cow-to-cow transmission is needed.

## RISK TO HUMANS

Farm workers, especially those in the milking parlor, comprise the majority of human cow H5N1 cases identified so far, some with confirmed infection resulting from a direct milk splash into the eye (6, 34). Various human ocular cell types contain receptors that can be bound by avian influenza viruses and can support virus replication (51, 52), potentially explaining the prevalence of eye infections. Given the high viral load in the milk of infected cows and the close contact between cows and workers, the milking parlor is an area of increased risk for cow–human transmission of H5N1. This is a wet, humid environment where milk splatter occurs throughout the milking process, especially during forestripping when milk is hand expressed, and the use of water hoses to clean areas may generate large droplets or aerosols containing viruses. The lack of eye or respiratory protection in milking parlors increases the risk of further human infections at this critical animal–human interface (Fig. 2).

Since milk from H5N1-infected cows can contain high levels of infectious virus, the USDA conducted surveys of commercially sold milk and milk products from across the country during April and June–July 2024. These studies revealed the presence of cow H5N1 nucleic acids in 20.2% and 17.4% of sampled products, respectively (53, 54). None of the sampled products tested positive for infectious virus based on egg inoculations, indicating that pasteurization can inactivate most of the H5N1 virus found in milk products. However, these data highlight the risk of consuming milk products that do not undergo thorough pasteurization.

### Gaps

Transmission of cow H5N1 from dairy farms to poultry farms has been observed in both Colorado and Michigan. The mechanism of the spread of the H5N1 B3.13 clade to poultry is largely unknown and is thought to be driven by shared workers across these two industries within an area, highlighting the need for increased surveillance of this population. As of 12 December 2024, at least 21 poultry workers have tested positive for cow H5N1, despite longstanding biosecurity measures (12, 35). The mechanism of their exposure remains unknown and no additional biosafety measures have been implemented.

## ROLE OF PRE-EXISTING IMMUNITY TO SEASONAL INFLUENZA INFECTIONS

Human cases of cow H5N1 have occurred in the context of pre-existing immunity to seasonal influenza viruses, as most people experience their first infection by age 5 (55). As of 12 December 2024, the reported human infections with cow H5N1 (H5N1 B3.13 clade) have been mild, which is in stark contrast to the severe disease observed in experimentally infected mice, ferrets, or cats (7, 42, 56–59). The discrepancy in disease outcome could be due to prior immunity. Studies examining the effects of pre-existing immunity against seasonal influenza viruses may help explain the mild symptoms experienced by those infected with cow H5N1. Ferrets with pre-existing immunity to the 2009 pandemic H1N1 virus strain (H1N1pdm09) were protected from severe symptoms and mortality after subsequent infection with cow H5N1 (60). In contrast, cow H5N1 caused severe symptoms and mortality in studies with immunologically naïve ferrets, including animals that were ocularly inoculated (57–60). Naïve ferrets developed systemic infections, with high viral titers in affected tissues, whereas pre-immune ferrets exhibited lower titers constrained to the respiratory tract. Analysis of human serum has detected circulating cross-reactive antibodies against H5N1 B3.13 clade in individuals born before 1970, although animal studies have suggested additional mechanisms of protection independent of circulating neutralizing antibodies (60, 61). While most human infections of 2.3.4.4b H5N1 clade have been mild, including H5N1 strains circulating since 2022, there is evidence of viral adaptation within humans, including evidence of the mammalian PB2 627K adaptation and changes within the receptor binding domain (62, 63). This underscores the pandemic risk of this virus, and every measure should be taken to curb the outbreak and limit the potential for further human adaptation.

## URGENT NEEDS

To contain the current outbreak, the following steps are needed:

- Longitudinal sampling of individual cows to assess the length of the infectious period and whether cows can become reinfected.
- Tracing of infected cows and animals using the same milking equipment to provide important data regarding transmission.
- Broader testing of dairy worker populations, as well as the individuals they interact with, which is critical to accurately assess the frequency of virus spillover and initiate timely responses.
- Robust biosecurity measures such as personal protective equipment (PPE; eye, respiratory protection, and hand washing), education of key populations about the virus and symptoms of infection, and disinfection of equipment moving between farms.
- Treatment of contaminated milk waste from dairy farms to inactivate the virus prior to disposal to ensure no livestock, pets, or peridomestic wildlife is further infected by it.
- Deployment of stockpiled H5 vaccines that can confer protection against the cattle 2.3.4.4b clade.

The widespread outbreak in cattle and frequent spillover into humans highlights the urgent needs for increased surveillance and mitigation efforts to combat the spread of cow H5N1.
